# Quantifying Key Environmental Determinants Shaping the Ecological Niche of Fruit Moth *Carposina sasakii* Matsumura, 1900 (Lepidoptera, Carposinidae)

**DOI:** 10.3390/insects17010109

**Published:** 2026-01-18

**Authors:** Ziyu Huang, Ling Wu, Huimin Yao, Shaopeng Cui, Angie Deng, Ruihe Gao, Fei Yu, Weifeng Wang, Shiyi Lian, Yali Li, Lina Men, Zhiwei Zhang

**Affiliations:** 1 College of Forestry, Shanxi Agricultural University, Jinzhong 030801, China; h1254730063@163.com (Z.H.); 17358681741@163.com (L.W.); 15698398062@163.com (H.Y.); shaopengcui@126.com (S.C.); gaoruihe1989@163.com (R.G.); yufei@sxau.edu.cn (F.Y.); wangwf2020@163.com (W.W.); 18234603390@163.com (S.L.); 18536761949@163.com (Y.L.); 2 Shanxi Dangerous Forest and Grassland Pest Inspection and Identification Center, Jinzhong 030801, China; 3 Lucile Packard Children’s Hospital at Stanford, Palo Alto, CA 94303, USA; andeng@stanfordchildrens.org

**Keywords:** peach fruit moth, MaxEnt, dispersal threshold, migration patterns, early-warning model

## Abstract

*Carposina sasakii* Matsumura is a significant lepidopteran pest in the Carposinidae family, inflicting substantial damage on stone and pome fruit trees such as jujube, peach, and apple. Using MaxEnt, we assessed the worldwide climatic suitability for *C. sasakii* and its key environmental drivers, evaluating how climate change impacts dispersal risks. Integrating global occurrence records with 37 environmental variables, the model (AUC = 0.982) quantitatively identifies July precipitation (prec7), minimum average temperatures in April and August (tmin4 and tmin8, respectively), and maximum average temperature in May (tmax5) as critical distribution determinants. Among these, prec7 exhibits the highest contribution (threshold approximately 370 mm). The current suitable habitat spans 10.39 × 10^2^ km^2^, concentrated predominantly in East Asia’s temperate monsoon zone (eastern China, the Korean Peninsula, and Japan) and southern North America. Under future climate scenarios, the high-emission pathway (SSP585) will reduce highly suitable areas, while moderately suitable zones expand coastward. In contrast, SSP370 projects a significant, albeit phased, habitat increase with a 19.61% growth rate. Precipitation regimes and extreme temperatures jointly regulate niche differentiation in *C. sasakii*, whose range shifts toward Southeast Asia and suboptimal regions in Europe and America, underscoring cascading climate change effects. These findings provide a scientific basis for transnational monitoring, early warning systems, and regional ecological governance.

## Simple Summary

This study investigates a significant fruit tree pest, *Carposina sasakii*, under global climate change. We quantified nonlinear responses of its distribution to precipitation, identifying July precipitation as a critical dispersal threshold. A “coastal contraction–inland transfer” migration model was developed, revealing synergistic effects of extreme temperature and precipitation on niche differentiation. The cross-border risk warning framework integrates stable East Asian sources with emerging Euro-American habitats to guide monitoring and management strategies.

## 1. Introduction

*Carposina sasakii* Matsumura, 1900 (Lepidoptea: Carposinidae), a destructive pest to stone and pome fruit trees, poses a significant threat to global fruit production. This species exhibits particularly severe impacts on northern China’s fruit agriculture, where it primarily damages *Ziziphus jujuba*, *Malus pumila*, and other commercially important stone and pome fruit trees. Initial larval feeding produces characteristic white film formations on fruit skin, leading to malformations colloquially termed, “monkey-head fruit.” Advanced infestation results in extensive internal tunneling with frass accumulation, rendering fruits commercially unviable. Documented infestation rates reach 40 to 70% in major apple-producing areas of northern China with the potential for complete fruit loss. The concealed larval stages within infested fruits facilitate transboundary dispersal through commercial fruit transportation networks, thereby exacerbating the species’ potential for ecological invasion. This mode of anthropogenic-mediated dispersal significantly elevates biosecurity risks across international agricultural systems [1,2,3].

The population dynamics and distribution patterns of *C. sasakii* demonstrate strong dependence on environmental parameters, with temperature, humidity, precipitation, and soil moisture collectively regulating developmental rates, overwintering success, and reproductive output [4]. Contemporary climate change has increased the frequency of extreme weather events, potentially altering the spatiotemporal distribution of suitable habitats and modifying the species’ invasive potential [5,6]. Systematic analysis of niche requirements and limiting factors is therefore critical for ecological risk assessment and mitigation.

Species distribution models (SDMs) provide essential tools for evaluating habitat suitability under climate change scenarios [7,8]. Among SDM algorithms, MaxEnt software (version 3.4.1) demonstrates particular utility for analyzing distribution patterns and environmental drivers, offering reliable prediction even with limited occurrence data [9]. This approach integrates multidimensional environmental variables (temperature, precipitation, soil moisture) to quantify niche requirements and identify distribution-limiting thresholds for *C. sasakii* [10]. Compared to conventional models, MaxEnt effectively discriminates among environmental variable contributions and simulates distributional shifts under future climate projections [11,12], thereby establishing a robust framework for understanding the species’ global ecological adaptations.

Recent climatic fluctuations and agricultural intensification have significantly altered *C. sasakii* distribution and impact patterns. To support effective pest management strategies, this study employs MaxEnt modeling with global occurrence records and high-resolution environmental data to (1) predict potential global distribution, (2) assess environmental factor importance, and (3) identify high-risk invasion zones. The results provide critical insights for developing targeted monitoring systems, early warning protocols, and international ecological governance, while informing adaptive management strategies for fruit tree pests experiencing climate change.

## 2. Materials and Methods

### 2.1. Acquisition and Processing of Geographic Distribution Data for C. sasakii

**Figure 1:**
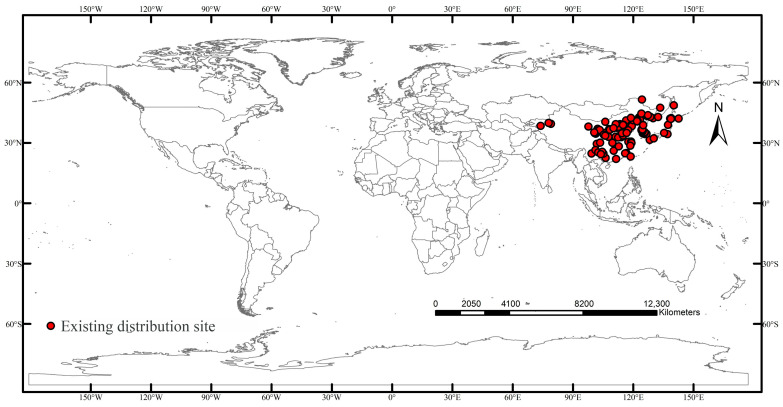
Global distribution of *Carposina sasakii* occurrence points.

Distribution data for *C. sasakii* were primarily obtained from the Global Biodiversity Information Facility (GBIF; https://www.gbif.org, https://doi.org/10.15468/39omei, accessed on 15 September 2025) [13], the European and Mediterranean Plant Protection Organization (EPPO; https://gd.eppo.int, accessed on 15 September 2025), and the relevant literature. Geographic locations lacking precise coordinates were georeferenced to the township level using the Chinese web mapping platform Ba Jiu Wang (https://bajiu.cn/ditu/?=40, accessed on 16 September 2025). Invalid and duplicate records were identified and removed in Microsoft Excel, resulting in a preliminary dataset of 139 distribution points. To mitigate potential overfitting caused by sample size and quality effects on model predictions [14], spatial thinning was performed using the Spatial Analyst tool in ArcMap 10.8.1. Since the environmental variable resolution was 5 arc-minutes, the rarefaction distance parameter was set to 5 km [15]. All adjacent redundant vertices within this threshold were removed, retaining only key nodes that significantly contributed to the geometry [14]. The thinned coordinates of *C. sasakii* were saved in CSV format following the “species name, longitude, latitude” convention, yielding a final dataset of 97 valid data points (Figure 1).

### 2.2. Environmental Data Acquisition and Processing

This study incorporated 37 environmental variables relevant to the life cycle of *C. sasakii*. Based on the insect’s biology, we selected temperature parameters (mean maximum and minimum temperatures), precipitation-related climate factors from April to September [16], and 19 bioclimatic variables.

Environmental data were sourced from the WorldClim climate database (https://www.worldclim.org, accessed on 17 September 2025) at a spatial resolution of five arc-minutes. Current environmental variables represented the 1971–2000 year baseline, while future projections covered four time periods: 2021–2040, 2041–2060, 2061–2080, and 2081–2100. Future climate data were derived from the Coupled Model Intercomparison Project Phase 6 (CMIP6). To evaluate the uncertainty in our projections, three representative CMIP6 models (CMCC-ESM2, BCC-CSM2-MR, and MIROC6) were used to generate predictions first. The spatial patterns and total projected suitable area (less than 495 Km^2^) for *Carposina sasakii* were basically consistent across models. Specifically, the results from the three models are identical under the current climate scenario. CMCC-ESM2 was selected for detailed analysis and presentation due to its accurate simulation of temperature, precipitation, and wind patterns, as well as its enhanced representation of terrestrial biogeochemistry, including expanded carbon pools, plant functional types, and nitrogen cycle dynamics [17,18]. These were closely corresponding to the climatic factors selected in this study. The Shared Socioeconomic Pathways (SSPs) were used to account for varying radiative forcing scenarios. Four SSPs were considered: SSP126 (low forcing, 2.6 W/m^2^), SSP245 (medium forcing, 4.5 W/m^2^), SSP370 (medium-high forcing, 7.0 W/m^2^), and SSP585 (high forcing, 8.5 W/m^2^) by 2100 [19]. Greenhouse gas emissions and ozone concentrations are the primary drivers of these radiative forcing levels [20].

**Figure 2:**
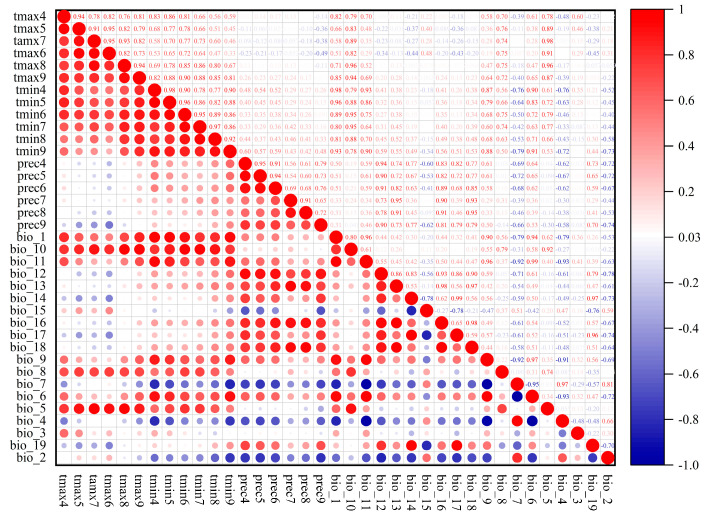
Pearson correlation analysis and correlation coefficients of 37 environmental variables. The figure displays pairwise correlations between variables, where the size of each circle is proportional to the statistical significance of the association. A larger circle indicates a stronger correlation.

Excessive environmental variables can compromise model stability and predictive accuracy due to autocorrelation and multicollinearity. Therefore, we screened variables to eliminate redundancy and improve model accuracy [21]. First, all occurrence points and environmental variables were imported into ArcMap 10.8.1 to extract environmental data for *C. sasakii,* followed by conversion from *.tif to *.asc format for modeling and analysis. Next, MaxEnt software was used to calculate the contribution rates of each variable, and variables with contribution rates below the 1% threshold were excluded from the final model [22]. Finally, correlation analysis was performed in IBM SPSS Statistics 27 (Figure 2). If the correlation coefficient |r| between two variables was ≥0.8, the variable with superior contribution was exclusively retained [23]. This process resulted in the selection of seven environmental variables for model construction (Table 1).

### 2.3. Using the MaxEnt Model to Predict Global Suitable Habitats for C. sasakii

#### 2.3.1. Parameter Settings

This study employed MaxEnt software to model the potential global distribution of *C. sasakii* under current and future climate scenarios. Distribution data (CSV format) and processed environmental variables (ASC format) were imported into the software. The following parameters were configured in the MaxEnt model: response curves and jackknife tests were enabled to assess environmental variable contributions to species distribution, and output format was specified as “Logistic” with file type “ASC” and replicate run type “Subsample” [24]. Random seed selection was activated to ensure unique initialization for each run. Data were partitioned with 25% allocated for testing and 75% for training. The model executed 10 replicate runs with identical parameter settings (maximum iterations = 5000) while maintaining default values for other parameters. The final output represented the averaged predictions across all replicates in ASC format. For future climate projections, all required environmental variables were placed in the projection layers directory/file prior to model execution [25].

**Figure 3:**
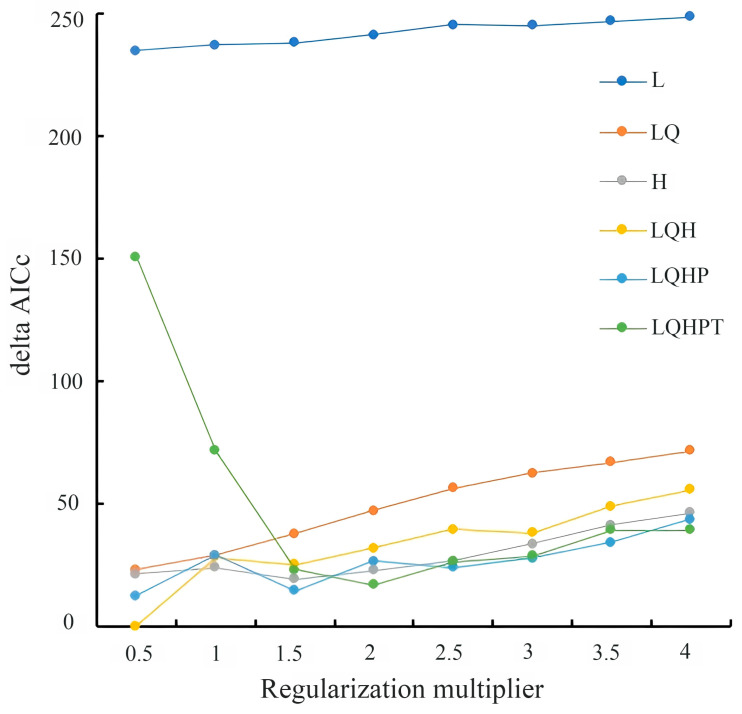
Parameter optimization results from the ENMeval R package (version 2.0.4). L, linear; Q, quadratic; H, hinge; P, product; T, threshold. L, LQ, H, LQH, LQHP, and LQHPT are different characteristic combinations. AICc: Corrected Akaike information criterion.

To optimize model performance and prevent overfitting, we utilized the “ENMeval” package in R to evaluate parameter combinations. Five feature classes were tested, linear (L), quadratic (Q), hinge (H), product (P), and threshold (T), with six combinations (L, LQ, H, LQH, LQHP, LQHPT) and eight regularization multipliers (0.5–4.0 in 0.5 increments). The best parameter combination was identified as the one yielding the smallest delta AICc [26]. For *C. sasakii*, the LQH feature combination with a regularization multiplier of 0.5 yielded optimal performance (Figure 3).

#### 2.3.2. Accuracy Assessment of the MaxEnt Model

Model accuracy was assessed using the receiver operating characteristic (ROC) curve, with the area under the curve (AUC) serving as the evaluation metric. Prediction accuracy was categorized as unsatisfactory (0–0.6), poor (0.6–0.7), average (0.7–0.8), good (0.8–0.9), and excellent (0.9–1) [27].

#### 2.3.3. Habitat Suitability Classification and Centroid Shift Analysis for *C. sasakii*

Using ArcMap 10.8.1, model outputs were reclassified via natural breaks into four suitability categories [28]: unsuitable distribution (≤0.1), low distribution (0.1–0.3), moderate distribution (0.3–0.6), and high distribution (≥0.6). Spatial distribution changes were quantified using centroid shift analysis, comparing current and projected suitable habitat centroid with the Centroid Changes Lines tool [29].

## 3. Results

### 3.1. Model Validation

**Figure 4:**
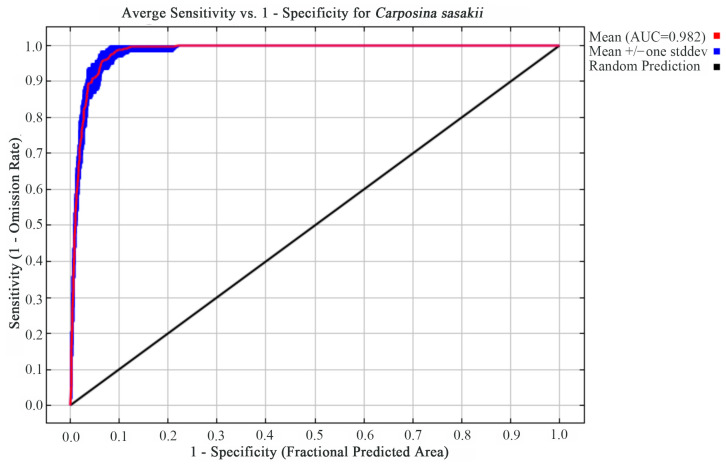
ROC curve of the MaxEnt model.

The MaxEnt model demonstrated excellent predictive performance, with training and test AUC values of 0.982 (Figure 4). The random prediction baseline AUC was 0.5. These results, combined with significant correlations between environmental variables and *C. sasakii* distribution, confirm the model’s reliability for ecological niche simulations and spatial pattern predictions.

### 3.2. Environmental Variable Contributions

The seven key variables influencing *C. sasakii* distribution, ranked by contribution rates, were prec7 (July precipitation), tmin8 (August minimum temperature), tmin4 (April minimum temperature), bio4 (temperature seasonality), bio15 (precipitation seasonality), bio19 (coldest quarter precipitation), and tmax5 (May maximum temperature) (Table 1).

The three most influential environmental variables affecting *C. sasakii* distribution were prec7, tmin8, and tmin4, collectively accounting for 69.4% of the total contribution rate. Permutation importance analysis identified tmin8, tmin4, and tmax5 as the most critical factors, representing 85.4% of total importance. Precipitation variables contributed 50.7% to the model (prec7 alone contributing 38%), while temperature factors accounted for 49.3%. These results demonstrate that precipitation exerts a slightly greater influence than temperature on *C. sasakii*’s potential distribution under current climatic conditions.

**Figure 5:**
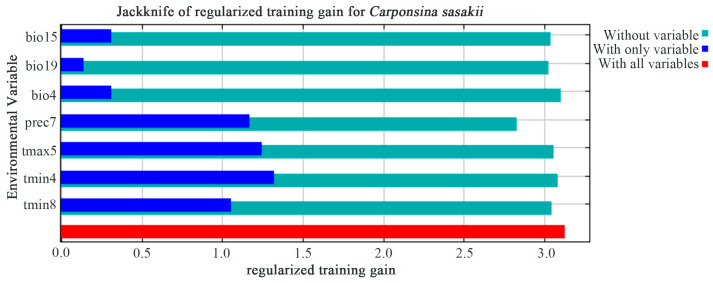
Jackknife test of variable importance showing regularized training gains for individual environmental factors.

Jackknife analysis (Figure 5) revealed that tmin4, tmax5, and prec7 contained the most biologically meaningful information for predictions. Notably, prec7 showed the smallest normalized training gain when excluded, demonstrating its unique predictive value [30]. These results establish prec7 as the primary limiting factor, followed by tmin4, tmin8, and tmax5. These results indicate that these variables contain more biologically meaningful information than others. In contrast, bio19 showed the lowest training gain when used independently, demonstrating its negligible contribution to the *C. sasakii* distribution model’s predictive accuracy. According to the “without variable” evaluation metric [30], variables with smaller regularized training gain values possess more unique information content. Among them, the normalized training gain value of prec7 is significantly lower than that of other variables, which further confirms our research: prec7 provides unique and crucial information for simulating the global habitat suitability of *C. sasakii*. This finding suggests prec7 captures ecological patterns not represented by other variables, establishing its fundamental importance in the model. The analysis identifies prec7, tmin4, tmax5, and tmin8 as key environmental determinants of *C. sasakii*’s current distribution.

### 3.3. Threshold Analysis of Dominant Environmental Variables in the Suitable Distribution Area of C. sasakii

**Figure 6:**
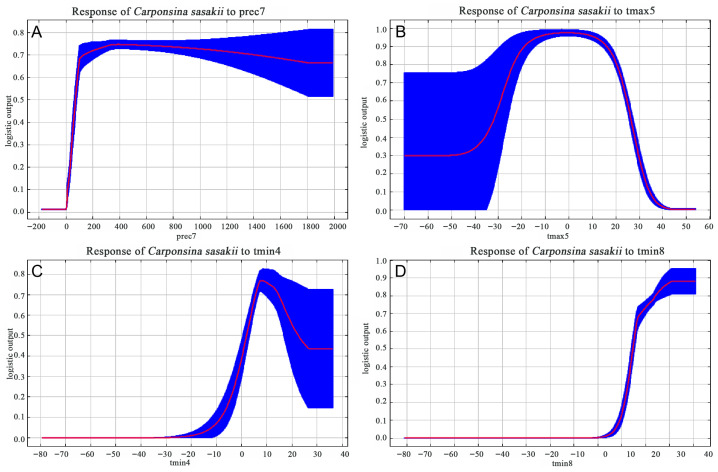
Response curves of dominant environmental factors for *C. sasakii*. (**A**) prec7 (July precipitation, mm); (**B**) tmax5 (May average maximum temperature, °C); (**C**) tmin4 (April average minimum temperature, °C); (**D**) tmin8 (August average minimum temperature, °C). Red represents mean value, blue represents Standard Deviation.

Response curve analysis (Figure 6) revealed the relationship between *C. sasakii*’s occurrence probability and four key environmental variables. For prec7, the optimal value was 370 mm, with survival probability < 0.5 at approximately 130 mm. Probability increased sharply with precipitation up to 370 mm, then gradually declined until 1800 mm, stabilizing >0.65 beyond this threshold. For tmax5, probability increased rapidly from −40 °C, peaking between −10 °C and 10 °C (optimal range), and then declined progressively up to 40 °C. Regarding tmin4, the optimal value was 8 °C. Probability remained <0.5 below 3 °C, increased from −20 °C to 8 °C, decreased from 8 to 25 °C, then stabilized at 0.44 above 40 °C. For tmin8, probability was <0.5 below 14 °C, peaked (0.88) at 31 °C, and remained stable above this threshold.

### 3.4. Prediction Results Under Current Climate Conditions

**Figure 7:**
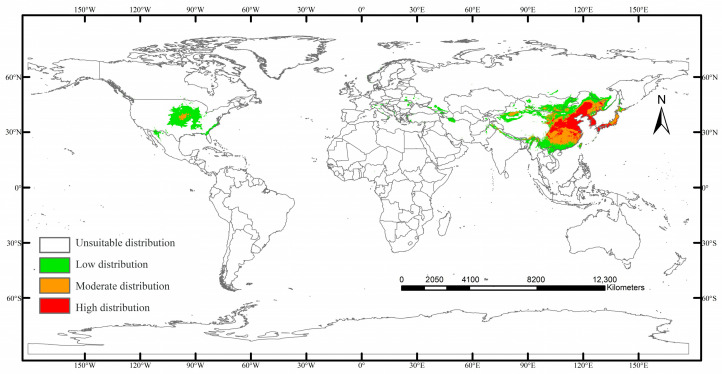
Potential global distribution of *Carposina sasakii* under current climate conditions.

The current suitable habitat distribution of *C. sasakii*, simulated using the MaxEnt model, demonstrates significant global regional differentiation in potential suitability (Figure 7). The core distribution is concentrated in eastern Asia, encompassing eastern China, Japan, the Korean Peninsula, southeastern Mongolia, and Russia’s Primorsky Krai. Secondary suitable areas occur in southern North America (e.g., central and southern United States), whereas European suitability remains limited. Globally, suitable habitats span 10.39 × 10^2^ km^2^, comprising high-suitability zones (2.02 × 10^2^ km^2^, 19.41%) in contiguous regions from northern to northeast China, the Korean Peninsula, and Japan’s Honshu Island. Moderate-suitability zones (2.67 × 10^2^ km^2^, 25.65%) can be found in patchy distributions throughout China’s Yangtze River basin, western Korean Peninsula coasts, and Texas, United States. This spatial pattern underscores the species’ adaptation to East Asian monsoon climates and North American temperate semi-humid zones.

### 3.5. Predicted Results Under Future Climate Conditions

Climate scenario simulations reveal dynamic shifts in the global potential habitat distribution of *C. sasakii*. While Southeast Asia persists as the core suitable region, total suitable habitat area demonstrates an overall decline from the present to years 2081–2100. Notably:

SSP585 scenario: Habitat area sharply contracts to 9.46 × 10^2^ km^2^ (2041–2060), representing the most severe reduction.

SSP370 scenario: Phased expansion occurs, reaching 11.71 × 10^2^ km^2^ (2021–2040, +12.66% vs. current) and 12.43 × 10^2^ km^2^ (2061–2080, +19.61%).

**Figure 8:**
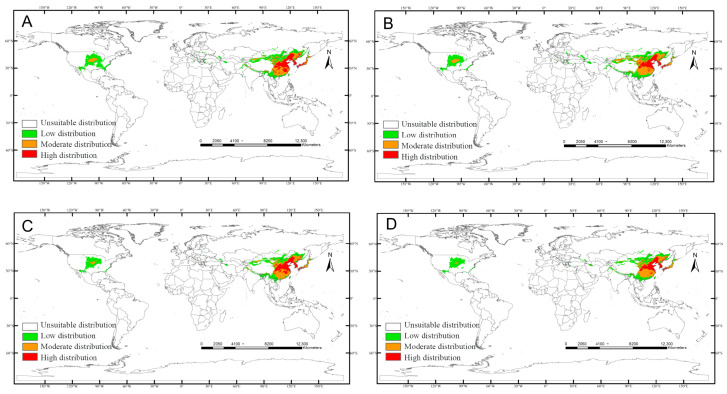
Predicted distribution of *Carposina sasakii* (years 2021–2100) under SSP126 scenarios: (**A**) 2021–2040, (**B**) 2041–2060, (**C**) 2061–2080, (**D**) 2081–2100.

Spatially, the United States’ central/southern moderate-suitability zone expands markedly under SSP370, while the European low-suitability zone fragmentarily increases (Greece, Italy, Romania, Ukraine). Scenario-specific differentiations emerge:

SSP126: Uniform contraction across all suitability tiers (Figure 8).

**Figure 9:**
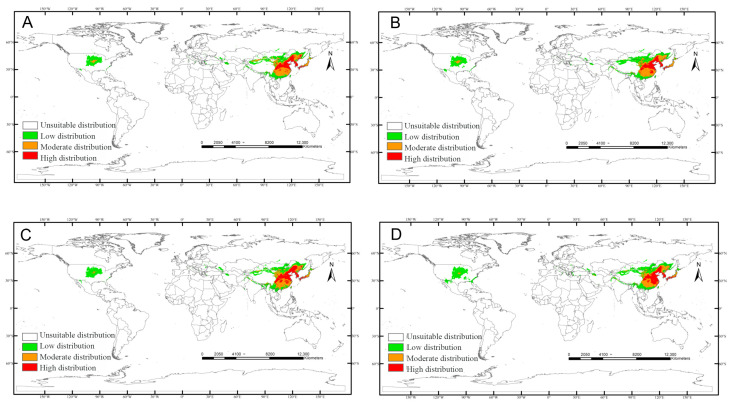
Predicted distribution of *Carposina sasakii* (years 2021–2100) under SSP245 scenarios: (**A**) 2021–2040, (**B**) 2041–2060, (**C**) 2061–2080, (**D**) 2081–2100.

SSP245: High/low-suitability area increases (low-suitability most pronounced), while medium-suitability shrinks (Figure 9).

**Figure 10:**
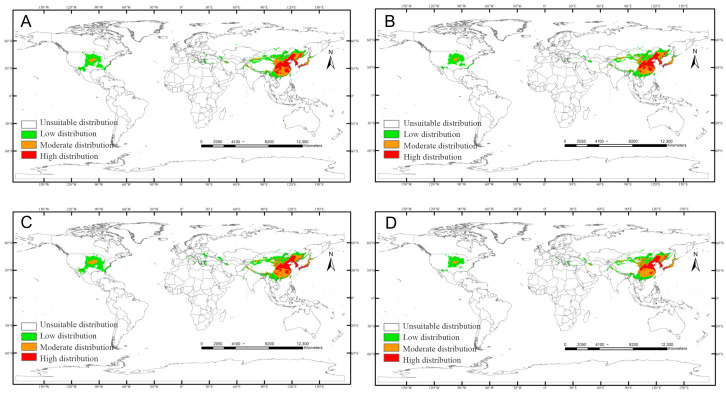
Predicted distribution of *Carposina sasakii* (years 2021–2100) under SSP370 scenarios: (**A**) 2021–2040, (**B**) 2041–2060, (**C**) 2061–2080, (**D**) 2081–2100.

SSP370: Medium-suitability expansion with concurrent high/low-suitability contraction (Figure 10).

**Figure 11:**
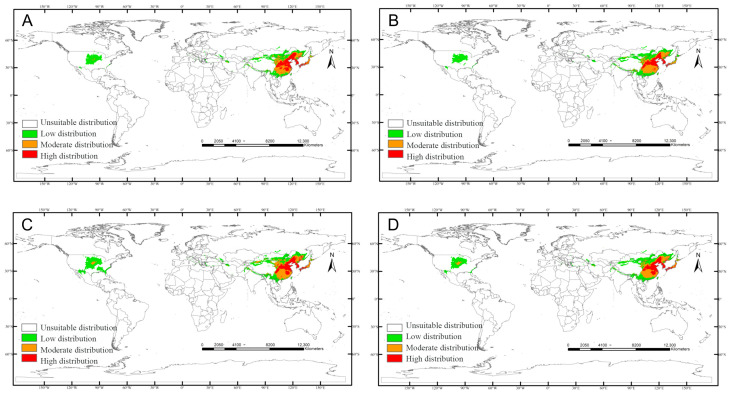
Predicted distribution of *Carposina sasakii* (years 2021–2100) under SSP585 scenarios: (**A**) 2021–2040, (**B**) 2041–2060, (**C**) 2061–2080, (**D**) 2081–2100.

SSP585: High-suitability suppression with marginal medium/low-suitability growth (Figure 11).

**Figure 12:**
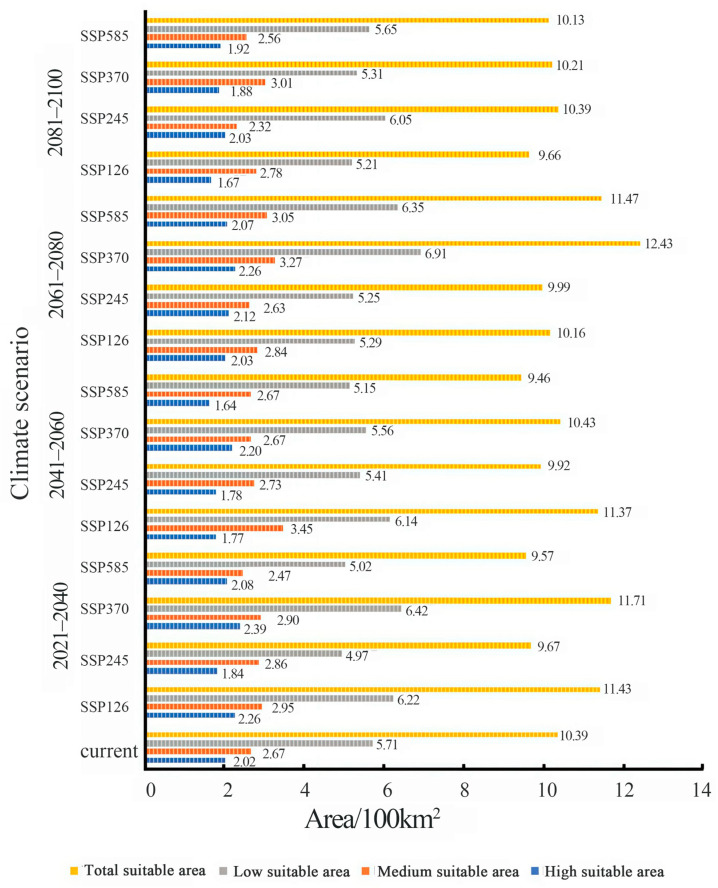
Comparative area projections for *Carposina sasakii* habitats under current and future climate scenarios.

The high suitable area, medium suitable area, low suitable area, and total suitable area projections for *C. sasakii* habitats under current and future climate scenarios are listed in Figure 12. These patterns demonstrate climate change’s nonlinear modulation of pest habitat structures.

**Figure 13:**
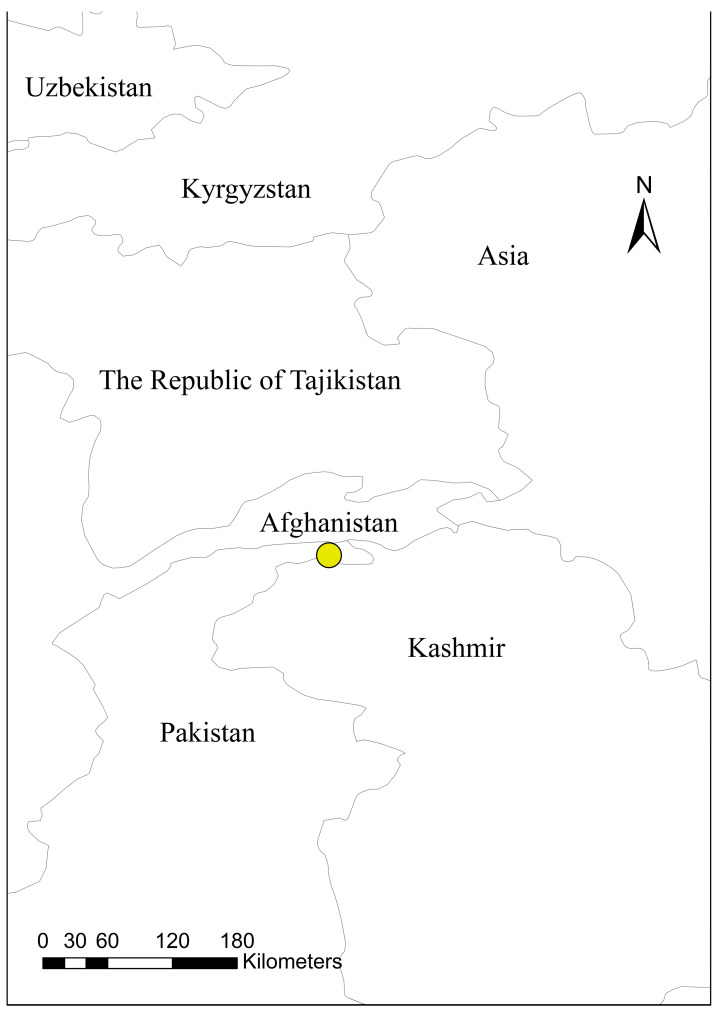
Geographic centroid of *Carposina sasakii*’s global potential habitat distribution.

Despite the profound influence of global climate change on species distribution patterns, our analysis reveals remarkable spatial stability in the distribution centroid of *C. sasakii*. Geographical coordinates in the current and future scenarios did not change (73.494546 E, 36.777818 N) (Figure 13). This stability stems from the species’ strict climatic requirements, particularly its niche conservatism concerning July precipitation (approximately 370 mm) and critical temperature thresholds (April/August minimum temperatures and May maximum temperature). While future climate scenarios project habitat modifications, including SSP585-induced contraction and SSP370-driven regional expansion, the Asian region persistently meets the species’ optimal hydro-thermal conditions, thereby anchoring its global distribution center. These findings suggest that, although climate change may reshape marginal distribution areas [31], climate-dependent species exhibit strong geographic inertia in their core ranges [32], with significant centroid shifts only occurring when climatic parameters surpass niche tolerance thresholds [33,34].

## 4. Discussion

This study employed the MaxEnt model to construct an ecological niche model for *Carposina sasakii*, systematically evaluating the evolution of its global suitable habitat under current and future climate scenarios. The objectives were to elucidate the driving mechanisms of climate change on *C. sasakii*’s dispersal risk and to propose corresponding control strategies. Analysis revealed that prec7, tmin4, tmin8, and tmax5 constitute dominant environmental factors limiting the insect’s suitable habitat distribution. The current global suitable habitat spans 10.39 × 10^2^ km^2^, with the core distribution area predominantly concentrated in the East Asian monsoon climate zone, encompassing eastern China, the Korean Peninsula, and the Japanese archipelago. Under the scenario of sustained carbon emission increases, simulations predict a polarized expansion pattern in suitable habitats for *C. sasakii*: expansion into Southeast Asia’s tropical monsoon regions and the formation of new suitable habitat patches along Europe’s Mediterranean coast and the central-eastern United States. This spatial expansion necessitates establishing a transcontinental coordinated pest prevention system with particular emphasis on biosecurity risks along international trade routes.

The Maximum Entropy (MaxEnt) model achieves superior predictive accuracy, compared to other models, through its capacity to establish highly complex functional relationships with observational data. This predictive performance primarily depends on three key parameters: the number of environmental variables, functional form, and regularization parameter [35]. Model validation using AUC values yielded an exceptional score of 0.982, confirming the model’s suitability for predicting *C. sasakii* habitat distribution.

Among environmental factors influencing *C. sasakii*’s distribution, July precipitation exerts the most significant impact. During the insect’s life cycle, precipitation during the peak growth period proves critical, with approximately 370 mm being optimal for population occurrence. As a fruit-boring pest in East Asia, *C. sasakii* enters its overwintering generation in spring, a critical period for population recovery and development. Due to effective accumulated temperature requirements, development is slow and population levels are low [16]. By July, when precipitation reaches 370 mm, adult emergence peaks, enhancing reproductive and dispersal abilities. Egg production and harm range expand, with increased hatching and larval survival rates. Previous studies found this to be the most active life stage, with the shortest generation time due to effective accumulated temperature [36], validating our analysis. Monitoring from spring and centralized control in July can efficiently manage populations by targeting adult activities, such as egg-laying at fruit outlets, achieving effective prevention and control. Precipitation affects insects directly via physical erosion and indirectly through plant chemical alterations, resulting in reduced larval survival rates, prolonged developmental periods, and modified plant surface properties that influence feeding behavior. Humidity directly impacts overwintering adult biology (lifespan, mating, and oviposition) and regulates developmental rates across all life stages. Crucially, during overwintering larval emergence, soil moisture and precipitation patterns become determinant factors for population size. Both excessive moisture/waterlogging and extremely low moisture conditions inhibit adult emergence [37,38]. Furthermore, precipitation patterns may indirectly limit *C. sasakii* geographic range by influencing host plant distribution [39].

MaxEnt model simulations reveal that *C. sasakii*’s current suitable habitat under present climate conditions primarily occupies the humid monsoon zone extending from East Asia to North America’s western coast, encompassing eastern China, the Korean Peninsula, Japan, southeastern Mongolia, Russia’s Far East, and the U.S. Pacific Northwest. This distribution strongly correlates with regions exhibiting consistent precipitation and stable humidity. Future climate scenarios demonstrate markedly divergent habitat evolution patterns: the SSP126 low-emission pathway predicts progressive global habitat reduction, demonstrating how climate mitigation measures constrain *C. sasakii*’s spread. Conversely, under the SSP585 high-emission scenario, while overall habitat area shows modest contraction, moderately suitable habitats expand into coastal low-elevation zones. This reflects climate warming’s dual effects: coastal soil moisture increases from glacial melt and sea-level rise create temporary larval emergence habitats [40], while global precipitation decline and aridification suppress adult emergence rates and reduce host fruit moisture, ultimately contracting high-suitability zones [38]. This simultaneous “contraction-substitution” dynamic illustrates climate change’s nonlinear restructuring of *C. sasakii*’s habitat architecture.

Analysis of *C. sasakii*’s current global distribution identifies potential suitable habitats in North America and Europe under both present and projected environmental conditions. Climate change impacts, particularly altered precipitation regimes combined with warming trends, are substantially modifying pest management approaches in North America. Precipitation exerts direct control over pest population dynamics through three primary mechanisms: soil moisture regulation, drought stress modulation, and host plant distribution modification. *C. sasakii* outbreak patterns show strong dependence on precipitation intensity and soil moisture availability, where optimal rainfall promotes population growth while excessive precipitation suppresses adult emergence rates. Concurrent warming trends have elevated annual temperatures, with summer peaks reaching 31 °C and spring averages near 8 °C, facilitating the moth’s rapid colonization of previously unaffected regions in North America and Europe. Post-establishment, the lack of natural predators may create vacant ecological niches [41], potentially amplifying infestation severity. To address these challenges, an integrated multidimensional management framework is proposed: (1) preemptive adjustment of monitoring and control zones based on projected habitat expansion trajectories, (2) implementation of biological control measures including natural enemy introduction and integrated pest management (IPM) to minimize broad-spectrum pesticide use while preserving ecosystem stability [42], (3) enhanced orchard sanitation through the removal of compromised trees and damaged fruit to disrupt favorable microhabitats, and (4) development of transnational monitoring networks and early warning systems to counter cross-ecosystem invasion risks [43].

The European Plant Protection Organization (EPPO) has designated *C. sasakii* as a quarantine pest based on its economic threat potential and transboundary dispersal risk [1,44]. Current research indicates its European habitat suitability remains relatively constrained, predominantly in marginally suitable areas, warranting critical evaluation of this regulatory classification. Two countervailing factors influence this assessment: while European establishment may be constrained by climatic thresholds and host plant availability, climate change could progressively expand suitable ranges, and international plant trade may facilitate accidental introductions [43]. Importantly, quarantine protocols serve dual functions: preventing localized outbreaks while providing non-tariff agricultural protection, even when initial habitat suitability appears limited. Consequently, maintaining quarantine status requires integrated analysis of climate projection models, host adaptation potential, and surveillance of cost–benefit ratios, informed by EPPO member states’ operational experience with border biosecurity and domestic monitoring systems.

Although the MaxEnt model offers advantages in simplicity, minimal sample requirements, and high prediction accuracy, it shares limitations with other niche prediction models [45]: (1) Overfitting occurs when distribution points are concentrated, leading to local environmental feature dominance and reduced generalizability [46]. Sparsity techniques mitigate spatial autocorrelation but cannot fully eliminate bias. (2) Species distribution is influenced by both abiotic and biotic factors. This study primarily used bioclimatic variables, excluding biological factors like host plant distribution and interspecific competition. Human activities also significantly impact *C. sasakii* populations. Current predictions focus on climate-driven geographical distribution, highlighting key climate characteristics [47]. Future work should incorporate host plant data, human factors, and additional variables for context-specific predictions.

## 5. Conclusions

This study systematically evaluates *Carposina sasakii*’s ecological niche dynamics under climate change using MaxEnt modeling. Current high-suitability habitats are concentrated in Asian monsoon regions, the Russian Far East, North American temperate zones, and the European Mediterranean coast, with projected southeastward shifts. July precipitation (prec7) demonstrates the strongest marginal effect on insect distribution, followed by threshold responses to April minimum temperature (tmin4), August minimum temperature (tmin8), and May maximum temperature (tmax5). These factors collectively regulate emergence phenology, reproductive success, and host fruit quality through cascading physiological mechanisms. Our findings elucidate climate-driven expansion pathways while informing biosecurity frameworks and orchard management strategies, particularly for optimizing surveillance in vulnerable regions.

## Figures and Tables

**Table 1:** Environmental variables affecting distribution.

No.	Climatic Variable	Contribution Rate/%	Permutation Importance/%
prec7	July precipitation	38	6.2
tmin8	August average minimum temperature	16.4	42.7
tmin4	April average minimum temperature	15	28.1
bio4	Temperature Seasonality (standard deviation × 100)	14.5	5.7
bio15	Precipitation seasonality	8.7	2
bio19	Precipitation in the Coldest Quarter	4	0.8
tmax5	May average maximum temperature	3.4	14.6

## Data Availability

The original contributions presented in this study are included in the article. Further inquiries can be directed to the corresponding author.

## Abbreviations

The following abbreviations are used in this manuscript:

*C. sasakii*	*Carposina sasakii*
SDMs	Species distribution models
CMIP6	Coupled Model Intercomparison Project Phase 6
CMCC-ESM2	Centro Euro-Mediterraneo sui Cambiamenti Climatici Earth System Model, version 2
BCC-CSM2-MR	Beijing Climate Center Climat System Model, version 2-Medium Resolution
MIROC6	Model for Interdisciplinary Research on Climate, version 6
SSPs	Shared Socioeconomic Pathways
AICc	Corrected Akaike information criterion
ROC	Receiver Operating Characteristic
AUC	Area Under the ROC Curve
